# Multifunctional ZnO Nanoparticles Synthesized Using *Spirodela polyrhiza* Extract: Characterization, Photocatalytic Activity, Antimicrobial Assessment, and In Silico Modeling

**DOI:** 10.1155/bca/5541535

**Published:** 2025-07-14

**Authors:** Azmat Ali Khan, Annu Yadav, Sudhakar Bansod, Azhar U. Khan, Nirmala Kumari Jangid, Mahboob Alam

**Affiliations:** ^1^Pharmaceutical Biotechnology Laboratory, Department of Pharmaceutical Chemistry, College of Pharmacy, King Saud University, Riyadh 11451, Saudi Arabia; ^2^Department of Chemistry, Banasthali Vidyapith, Banasthali 304022, Rajasthan, India; ^3^Fluoro-Agro Chemicals Department, CSIR-Indian Institute of Chemical Technology, Hyderabad 500007, India; ^4^School of Life and Basic Sciences, Jaipur National University, Jaipur 302017, Rajasthan, India; ^5^Department of Safety Engineering, Dongguk University, 123 Dongdae-ro, Gyeongju 780714, Gyeongbuk, Republic of Korea

**Keywords:** antimicrobial, biogenic synthesis, molecular docking, *Spirodela polyrhiza*, ZnO NPs

## Abstract

This study investigates the green synthesis of zinc oxide nanoparticles (ZnO NPs) using the aqueous extract of the aquatic plant *Spirodela polyrhiza* (greater duckweed) and evaluates their multifunctional properties. The ZnO NPs were synthesized via a sustainable method and characterized using UV-visible spectroscopy, TEM, FESEM, EDX, FTIR, and XRD analyses. UV-visible spectroscopy confirmed the formation of ZnO NPs with a characteristic absorption peak at ∼349 nm. TEM and FESEM analyses revealed spherical and nonspherical particles ranging from 20 to 70 nm. The antimicrobial activity of ZnO NPs was assessed against three bacterial strains (*Escherichia coli*, *Staphylococcus aureus*, and *Bacillus subtilis*) and three fungal strains (*Aspergillus niger*, *Penicillium chrysogenum*, and *Candida albicans*). Notably, *B. subtilis* showed a maximum inhibition zone of 18 mm at 100 mg/mL, while *A. niger* exhibited the highest antifungal response with a zone of 22 mm and an activity index (AI) of 1.15, indicating comparable or superior activity to ketoconazole at higher concentrations. Molecular docking simulations using the crystal structure of *B. subtilis* YmaH (Hfq) protein (PDB ID: 3HSB) revealed strong noncovalent interactions with Zn atoms of the NPs, particularly involving HIS57 and LEU26 residues. Additionally, ZnO NPs demonstrated a noteworthy photocatalytic degradation (90.4%) of methylene blue dye under sunlight exposure. These results highlight the potential of *S. polyrhiza*-mediated ZnO NPs for use in antimicrobial therapies and environmental remediation applications.

## 1. Introduction

The world's growing population puts pressure on food production, requiring increased agricultural production and crop yields. Plant diseases can cause severe yield losses, ranging from 20% to 40% [1, 2]. Traditional methods often rely on pesticides and fungicides, but there are growing concerns about their impact on human health and the environment [1]. This has led to a search for sustainable alternatives. Nanotechnology shows promise as a potential solution due to its unique properties and potential benefits to agriculture. Nanotechnology, the manipulation of materials at the microscopic scale of a billionth of a meter, holds immense potential to transform agriculture. Nanoparticles (NPs), with their unique properties due to their high surface area, offer exciting possibilities for addressing biotic stresses caused by plant pathogens [1, 3]. Research suggests that NPs can be significantly more absorbent than their bulk counterparts, potentially requiring 15–20 times less material [4]. This translates to a substantial decrease in the hazardous chemicals often employed in the treatment of plant diseases. Furthermore, the field of nanotechnology is embracing green synthesis techniques. This eco-friendly approach utilizes natural resources to produce NPs, minimizing the reliance on harsh chemicals. Globally, researchers are actively investigating the effects of NPs on agricultural productivity, plant development, and the extent to which they fight diseases in plants [1, 5]. Other NPs, such as gold NPs (Au NPs) and silver NPs (Ag NPs), have been successfully synthesized using *Allium cepa* (red onion) peel extract and *Solanum tuberosum* (potato) peel extract, respectively. The Au NPs exhibited significant antibacterial and antifungal activities, along with efficient inhibitory effects on acetylcholinesterase (AChE) and butyrylcholinesterase (BChE) enzymes. These spherical Au NPs, ranging in size from 6.08 to 54.20 nm, demonstrated promising therapeutic potential, highlighting the advantages of utilizing plant extracts in NP synthesis [6]. Similarly, the Ag NPs produced were small, stable, and showed significant antibacterial activity against multiple bacterial strains, including *Escherichia coli*, *Pseudomonas aeruginosa*, and *Staphylococcus aureus*. Additionally, the study reported cytotoxic effects against various cancer cell lines, further emphasizing the biomedical potential of plant-derived NPs [7]. Moreover, the utilization of apricot (*Prunus armeniaca* L.) kernel and pulp in the synthesis of bioactive compounds has been explored due to the fruit's rich composition of polyphenols, fatty acids, and essential minerals. These bioactive compounds have found applications in the food, pharmaceutical, and cosmetics industries, underscoring the broader impact of plant-based materials in diverse fields [8]. The potential of these compounds to function as reducing agents and stabilizers in the synthesis of ZnO NPs is noteworthy. In addition, the presence of these bioactive compounds can increase the surface area of ZnO NPs and reduce the recombination rate of photogenerated electron-hole pairs, thereby enhancing their photocatalytic activity [9, 10]. Plants require zinc, an essential micronutrient, for a variety of functions. Zinc is vital for enzyme activity, maintaining membrane integrity, and seed development [11]. Recent studies have shown that zinc oxide NPs (ZnO NPs) can improve agricultural yields and crop yields [12]. Studies reveal that treating peanut stem and root development with nanoscale zinc oxide [13]. Compared to other options, ZnO NPs appear to be beneficial for plant growth, less harmful to plants and helpful soil microbes, and have increased resistance to infections [14, 15]. Furthermore, ZnO NPs have also shown effectiveness as antibacterial agents [12]. According to estimates, the world produces between 550 and 33,400 tons of ZnO NPs annually, making it the third most extensively used nanomaterial [16, 17]. The environmental content of ZnO NPs is approximately 3.1–31 μg/kg in soil and 76–760 μg/L in water [18].

ZnO NPs have been shown to have good antibacterial properties against foodborne pathogens found in poultry [19]. The potential of ZnO NPs for developing different strategies to combat foodborne diseases has been demonstrated. ZnO NPs derived from various extracts of natural resources have been shown to have a wide range of beneficial applications in biological systems. These applications include antimicrobial activity, antioxidant properties, biomedical applications, antiparasitic effects, photocatalytic activity, and even antiskin cancer properties [20–25]. Various aquatic species have been employed for the green synthesis of ZnO NPs, as outlined in Table 1. The resulting NPs have been extensively characterized and compared. In this study, the potential of a common aquatic plant, *Spirodela polyrhiza*, for the synthesis of ZnO NPs was explored. *Spirodela polyrhiza* is shown to be a viable and environmentally friendly option for producing ZnO NPs, advancing the field of green nanotechnology. Using *S. polyrhiza* extract as a reducing and stabilizing agent eliminates the need for harsh chemicals, making NP production less harmful to the environment. Furthermore, the use of in silico modeling in this study provides an essential understanding of the interactions between ZnO NPs and biological targets. This knowledge has the potential to enhance the advancement of targeted drug delivery systems and antibacterial treatments. The findings have substantial suggestions for several biomedical fields, including wound healing, tissue engineering, and infection management. The present findings demonstrate that *Spirodela polyrhiza* can effectively mediate the formation of ZnO NPs, offering a sustainable and eco-friendly approach to NP production. However, numerous studies have efficiently synthesized ZnO NPs using different plant resources, highlighting the feasibility and promise of this ecologically conscious approach [32–40]. This research builds upon previous research by investigating green synthesis methods for ZnO NPs. Their antimicrobial properties were then evaluated, and molecular docking simulations were used to gain a deeper understanding of their inhibitory mechanisms.

Although several plant-based extracts have been employed in the green synthesis of ZnO NPs, the use of *Spirodela polyrhiza*, a fast-growing aquatic plant, remains underexplored. This study investigates the biosynthesis of ZnO NPs using environmentally friendly methods, capitalizing on the ability of plants to produce NPs and reducing dependence on hazardous and expensive chemical reagents. Furthermore, the combination of detailed antimicrobial profiling, photocatalytic degradation analysis, and molecular docking against the *Bacillus subtilis* YmaH protein (PDB: 3HSB) has not been reported to our knowledge, adding an innovative in silico perspective to green nanomaterial research.

## 2. Materials and Methods

### 2.1. General

All chemicals were purchased from commercial sources (Merck India Ltd) and used as received without further purification. UV-visible (UV–vis) spectra were recorded on a Shimadzu UV-1800 spectrophotometer (Japan) with a resolution of 1 nm between 200 and 800 nm. Transmission electron microscopy (TEM) with a TECNAI G-20 instrument and scanning electron microscopy (SEM) with a Nova nano FE-SEM 450 FEI instrument were used to determine the size and morphology of ZnO NPs (ZnO NPs). Fourier transform infrared (FTIR) spectroscopic analysis of the extracts was performed on a Perkin Elmer Spectrum 2000 FTIR spectrometer using KBr particle technology in the wavenumber range of 4000–400 cm^−1^. Energy dispersive x-ray spectroscopy (EDX) analysis confirmed the presence of ZnO in the samples.

### 2.2. Preparation of Extract

The greater duckweed (*Spirodela polyrhiza*) displays a range of sizes and shapes. It has developed adaptations such as flat, floating fronds that enable it to stay close to the water's surface for maximum exposure to sunlight. Although the majority of reproduction takes place asexually by vegetative propagation, there is also the possibility of sexual reproduction under certain circumstances. Their typical habitat preferences tend to favor calmer waters, while particular species may be able to withstand somewhat more turbulent circumstances.

To isolate greater duckweed (*Spirodela polyrhiza*) from the water pond of National University, Jaipur, the specimens were removed and washed thoroughly with fresh water and then filtered using standard filter paper. Subsequently, the washed samples were dried at room temperature and finely powdered.

A 5.0 g portion of the dry powder was then boiled in 250 mL of deionized water in a round-bottom flask for 45 min, after which the solution was permitted to reach ambient temperature. The solution obtained was subjected to filtration using Whatman filter paper in order to achieve purification of the extract. Subsequently, the resultant filtrate was kept under cold conditions to facilitate further analysis.

### 2.3. Biosynthesis of ZnO NPs

*Spirodela polyrhiza* extract (20 mL) was added to a 250 mL conical flask, followed by the gradual addition of 80 mL of 0.01 M zinc acetate solution while stirring continuously. The temperature was maintained between 50°C and 60°C throughout the reaction. The reaction mixture showed a pale-yellow transition and produced a cream-colored precipitate, which was identified as zinc hydroxide. Subsequently, the reaction mixture was allowed to stand for 30 min to ensure completion of zinc hydroxide reduction, and then centrifuged at 15,000 rpm for 10 min. The resulting residue was dried under vacuum at 40°C–50°C and stored for subsequent analysis. The formation of ZnO NPs was indicated by the observed change in the solution's color, transitioning from a light-yellow hue to a cream tint. This formation was further confirmed by UV–vis spectroscopy, which showed a peak at 349 nm. The appearance of this absorption peak is attributed to the electron transition from the valence band to the conduction band, and it is not associated with surface plasmon resonance (SPR), which is characteristic of metallic particles. As noted by the Motelica group [41], such errors are frequently found in the literature.

### 2.4. Characterizations of ZnO NPs

As-prepared ZnO NPs using greater duckweed extract were monitored with a UV–vis spectrophotometer (Shimadzu, USA) in the 200–800 nm wavelength range. The NPs were structurally characterized by infrared spectroscopy using FTIR (Perkin Elmer 1750) to determine the functional groups present in the ZnO NPs. Further characterization was performed using x-ray diffraction (XRD), EDX, SEM, and TEM. SEM imaging and TEM imaging jointly confirmed the spherical morphology of ZnO NPs with a size distribution in the 20 nm range. XRD using Cu Kα radiation helps in obtaining XRD patterns of NPs.

### 2.5. Antibacterial Activity Study

The antibacterial activity was evaluated using the standard agar well diffusion method [42]. ZnO NPs were dispersed in sterile distilled water as the primary solvent with sonication for 15 min to ensure homogeneity, and test solutions were prepared at concentrations of 25, 50, 75, and 100 mg/mL in the same solvent. Nutrient agar (NA) plates were inoculated with standardized bacterial suspensions (adjusted to 1 × 10^8^ CFU/mL) of *Escherichia coli*, *Staphylococcus aureus*, and *Bacillus subtilis*. Wells of 6 mm diameter were aseptically prepared in the inoculated agar plates, into which 30 μL of each test solution was introduced. Streptomycin, dissolved in sterile distilled water at the same concentration, served as a positive control. All plates were incubated aerobically at 37°C for 24 h, after which the diameters of inhibition zones (IZD) were measured in millimeters (mm) from the edge of the well to the edge of the clear zone.

The activity index (AI) was calculated to compare the efficacy of the test samples with the standard antibiotic (streptomycin) using the formula:

(1) $$\begin{array}{c} \text{AI}=\frac{\text{IZD of sample}}{\text{IZD of standard}}\left(\text{streptomycin}\right). \end{array}$$

### 2.6. Antifungal Activity Study

The antifungal activity of the ZnO NPs was evaluated against *Aspergillus niger*, *Penicillium chrysogenum*, and *Candida albicans* using the well diffusion method on potato dextrose agar (PDA). For the assay, the NPs were dispersed in sterile distilled water with sonication for 15 min to ensure homogeneity. Test solutions were then prepared at concentrations ranging from 25 to 100 mg/mL using sterile distilled water as the solvent. Ketoconazole, also dissolved in sterile distilled water at equivalent concentrations, served as the reference antifungal agent. Fungal spore suspensions were adjusted to 1 × 10^8^ spores/mL using a hemocytometer and uniformly spread on PDA plates. Wells (6 mm diameter) were aseptically prepared in the agar and loaded with 30 μL of each test solution. The plates were incubated at 28°C for 48 h. After incubation, the diameters of the IZD were measured in millimeters, from the well edge to the colony margin. Experiments were performed in triplicate to ensure reproducibility. The AI was calculated as the ratio of the sample's IZ to that of the ketoconazole control (Equation (1)).

### 2.7. Photocatalytic Experiments

The photocatalytic activity of synthesized ZnO NPs was evaluated by assessing their ability to degrade methylene blue (MB) under direct solar irradiation. Before the photocatalytic experiments, ZnO NPs (0.6 g/L) were dispersed in an MB dye solution (10 μM, 150 mL) [10]. The mixture was stirred continuously in the dark for 60 min, followed by a 45-min equilibration period. Subsequently, aliquots (5 mL) were withdrawn from the reaction solution and filtered to remove ZnO NPs. The concentration of MB in these aliquots was then determined using a UV–vis spectrophotometer at a wavelength of 665 nm. The initial MB concentration was denoted as *C*_0_. The photocatalytic reaction mixture (MB dye and ZnO NPs) was exposed to sunlight, and the MB concentration (*C*_*t*_) was monitored at intervals of 0, 30, 60, 90, 120, and 150 min. The photocatalytic efficiency of ZnO NPs in degrading MB was calculated using the following equation:

(2) $$\begin{array}{c} \text{Photocatalytic Efficiency }\left(\%\right)=\left[\frac{C_{0}-C_{t}}{C_{0}}\right]\times 100, \end{array}$$

where *C*_0_ and *C*_*t*_ represent the initial and final MB concentrations at a specific time, respectively.

### 2.8. Molecular Docking

The crystal structure of the specific protein receptor YmaH (Hfq) from *Bacillus subtilis* bound to an RNA aptamer (PDB ID: 3HSB) was obtained from the RCSB PDB repository, based on literature demonstrating the potent inhibitory capacity of protein YmaH against target bacteria. The structure of ZnO NPs was constructed using Material Studio (BIOVIA, 2014) using crystallographic data of ZnO. These ZnO NPs were then subjected to a series of minimization and optimization procedures and then saved in PDB format. Following optimization, the structures of the prepared receptor and ZnO NPs were uploaded to a web-based molecular docking platform utilizing AutoDock Vina for pose prediction [43]. The identified docked complexes exhibiting the most favorable binding energies were selected for further investigation of noncovalent interactions using Discovery Studio [44].

## 3. Results and Discussion

In the last decade, several papers reported on the biosynthesis of ZnO NPs from plants [45, 46]. ZnO-NPs were synthesized via a green approach utilizing aqueous extract as a stabilizing agent. A comprehensive literature review informed this methodology for biosynthesized NP synthesis. The final formation of ZnO NPs is identified based on spectral (UV–vis, EDS, FTIR, SEM, and TEM). The experimental conditions used in this study, such as the temperature range, reagent concentrations, and use of greater duckweed extracts, were critical for the successful biosynthesis of ZnO NPs. The selected conditions were consistent with those reported in the literature mentioned above for similar biosynthetic processes, enhancing the reliability of this method. For example, a temperature range of 50°C–60°C was often employed to promote the formation of NPs while preventing the decomposition of organic components in the extract. The use of green nanomaterials, such as those synthesized from greater duckweed extracts, represents a promising alternative to conventional methods that often rely on toxic chemicals. Green synthetic methods are advantageous due to their environmentally friendly properties that reduce the environmental impacts associated with NP production. By utilizing plant extracts, this approach not only minimizes the use of hazardous substances but also provides a cost-effective and sustainable route to NP production. The present results contribute to the growing body of evidence supporting the effectiveness of green synthetic methods. ZnO NPs produced using *Azolla* extracts exhibit desirable properties and show potential for further applications in various fields such as environmental remediation and healthcare.

### 3.1. Characterization of Synthesized ZnO NPs

#### 3.1.1. UV–Vis Spectroscopy

**Figure 1:**
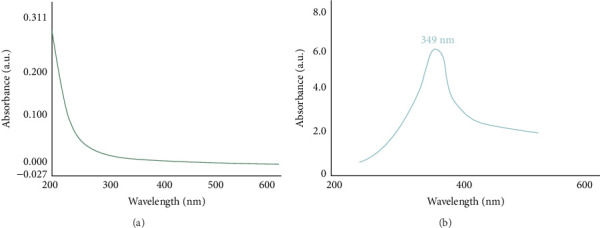
UV–vis spectra of (a) greater duckweed extract and (b) ZnO NPs obtained using greater duckweed.

Several studies have reported that biosynthesized ZnO NPs typically exhibit UV–vis absorption spectra in the range of 200–800 nm [46]. UV–vis spectroscopy was employed to characterize both the extract and the synthesized NPs (Figures 1(a) and 1(b)). The NPs exhibit a distinct absorption peak centered at 349 nm (Figure 1(b)). Since ZnO is a semiconductor, the prominent absorption peak may be more precisely ascribed to electronic transitions occurring in the band gap of the material, rather than SPR. This interpretation is supported by the Motelica research group, discussing band gap transitions in ZnO NPs [41]. This peak marks the successful biosynthesis of ZnO NPs and their stability in suspension. The synthesized ZnO NP appeared as a light brown powder, consistent with the UV–vis spectral data. The observed absorption peak at 349 nm is indicative of small NP formation, as confirmed by the UV–vis spectrum analysis.

#### 3.1.2. FTIR Analysis

FTIR spectroscopy was used to identify the functional groups present on the synthesized ZnO NPs that served as a protective capping agent. In the FTIR spectrum, prominent absorption bands were observed at 3433, 1640, 1555, 1413, and 486 cm^−1^. While the peaks at 1413 and 1556 cm^−1^ are characteristic of acetate ions, their presence is expected due to the use of zinc acetate as a precursor [47]. The remaining peaks indicate the presence of hydroxyl groups (3433 cm^−1^), carbonyl groups (1640 and 1555 cm^−1^), aromatic C=C bonds (1413 cm^−1^), and Zn-O stretching vibrations (486 cm^−1^), confirming the formation of ZnO NPs. Additionally, the presence of –NH groups (3339 cm^−1^) in the extract spectrum suggests that functional groups within the greater duckweed may play a role in the synthesis process. The absorption peak at 1635 cm^−1^ indicates the presence of -C=C- bonds within the aromatic ring.

**Figure 2:**
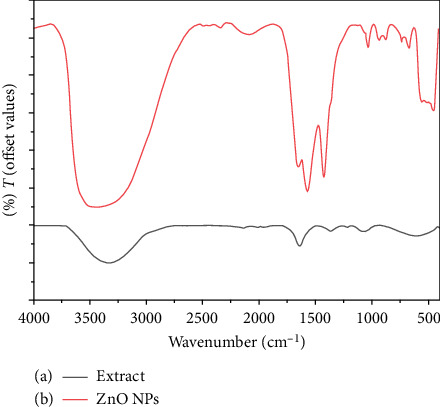
(a) FTIR of extract, (b) FTIR of ZnO NPs using the extract of greater duckweed.

These observations, together with the presence of –NH groups identified in the extract spectrum (3339 cm^−1^, Figure 2(a)), suggest that functional groups within the greater duckweed may play a dual role during the synthesis. Furthermore, the absorption peak observed at 1635 cm^−1^ indicates the presence of -C=C- bonds within the aromatic ring. FTIR spectroscopy (shown in Figure 2(b)) was used to identify potential plant-derived compounds responsible for the formation and stabilization of ZnO NPs synthesized using extracts. These compounds may act as reducing and capping agents, promoting the formation and stabilization of ZnO NPs. Various other moderately strong bands are discernible in the spectrum, corresponding to –C=C– stretching (typical of flavanones), a broad peak representing –N–H– stretching (associated with amides), and cyclic CH2 stretching (representing aliphatic compounds group). Notably, the prominent band observed at 428 cm^−1^ confirmed the formation of ZnO NPs. The presence of these unique peaks in the FTIR spectrum of ZnO NPs indicates its dual function as a green reducing agent and stabilizer.

#### 3.1.3. XRD Analysis

**Figure 3:**
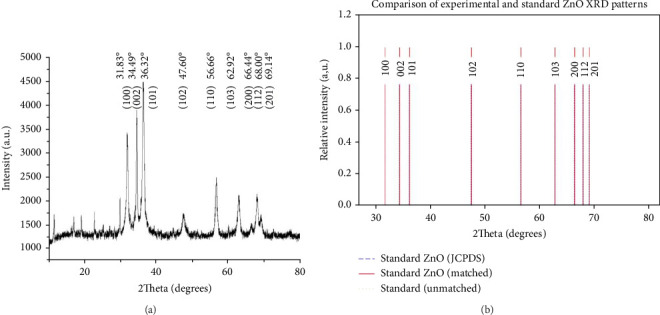
XRD Analysis of ZnO NPs. (a) Experimental XRD pattern of as-synthesized ZnO NPs, showing characteristic peaks indexed to the hexagonal wurtzite structure. (b) Comparative plot of observed experimental peaks (red solid lines) against standard ZnO JCPDS data (blue dashed lines), confirming ZnO formation.

Figure 3 shows the XRD pattern of ZnO NPs synthesized using greater duckweed extract. Diffraction peaks were observed at 2*θ* values 31.83°, 34.49°, 36.32°, 47.60°, 56.66°, 62.92°, 66.44°, 68.00°, 69.14°, 72.63°, and 77.03°, which were assigned to (100), respectively, and (002), (101), (102), (110), (103), (200), (112), and (201) as crystal planes. The XRD analysis confirms the successful synthesis of ZnO NPs with a wurtzite crystal structure, evidenced by the strong correlation between most observed peaks and ZnO JCPDS data (JCPDS Card No. 36–1451), which aligns with the wurtzite crystal structure of ZnO NPs reported in the existing literature [48]. However, the minor peaks at 72.63° and 77.03°, observed at very low intensities, do not match standard ZnO patterns and may indicate trace impurities or secondary crystalline phases.

#### 3.1.4. SEM

**Figure 4:**
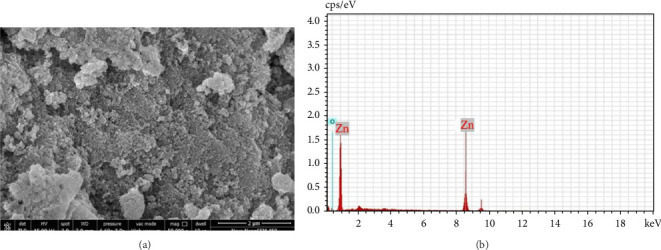
(a) SEM images of ZnO NPs. (b) EDS image of ZnO NPs.

High-resolution SEM was used to characterize the morphology of the synthesized ZnO NPs. The resulting micrograph (Figure 4(a)) provides observation into the distribution and surface characteristics of the NPs. The image shows a well-dispersed population of ZnO NPs. However, closer inspection at higher magnification revealed that the NPs assemble, indicating some degree of agglomeration. This phenomenon may be attributed to the attractive van der Waals forces promoting aggregation between spherical ZnO NPs.

#### 3.1.5. EDS

EDS analysis confirmed the presence of zinc (Zn) and oxygen (O) in the synthesized NPs. The corresponding EDS spectrum (Figure 4(b)) shows the characteristic x-ray emission peaks of these elements. The vertical axis represents the intensity of the detected X-rays (counts), and the horizontal axis represents the energy of the emitted X-rays (keV). The prominent peaks observed at approximately 8 keV correspond to the characteristic Kα and Kβ emission lines of zinc (Zn). In contrast, the peaks observed around 1 keV are associated with the Lα and Lβ emission lines of zinc. For oxygen (O), the peak around 0.5 keV corresponds to the Kα emission line. These observations strongly suggest the presence of ZnO in the sample. Quantitative analysis of the EDS spectra revealed an average mass composition of approximately 76.53% Zn and 14.21% O. This composition deviates from the theoretical mass percentages of ZnO, which are approximately 80.24% Zn and 19.75% O. Despite this discrepancy, the data indicates that ZnO is present in the sample, with the observed elemental ratio suggesting minimal impurities within the detection limits of the EDS analysis. In summary, EDS analysis confirms the presence of zinc and oxygen in the NPs, with strong peaks corresponding to these elements and their respective number ratios. This analysis supports the identification of the elemental composition of the synthesized NPs. The semi-quantitative ratios of these elements confirm the presence of Zn and O in the sample, supporting the intended composition of the NPs.

#### 3.1.6. TEM

**Figure 5:**
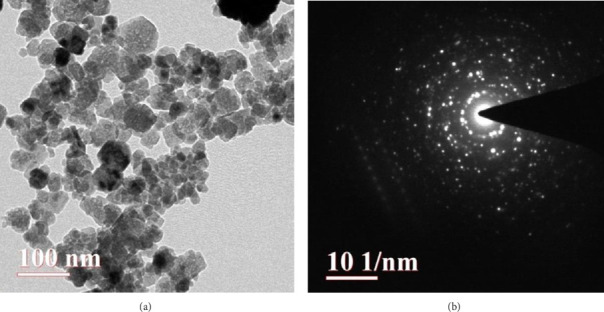
(a) TEM images of ZnO NPs. (b) SAED image of ZnO NPs.

TEM analysis revealed that the size of the NPs ranged from 20 to 70 nm. Specifically, ZnO NPs within the size range of 20–30 nm exhibited a crystalline structure, as evidenced by the analysis (Figure 5(a)). The selected area electron diffraction (SAED) pattern (Figure 5(b)) exhibits well-defined rings. This observation confirms the crystalline nature of ZnO NPs. Each ring in the pattern corresponds to electron diffraction from a specific crystallographic face within the ZnO lattice.

### 3.2. Analysis of Antibacterial and Antifungal Activity of ZnO NPs

**Figure 6:**
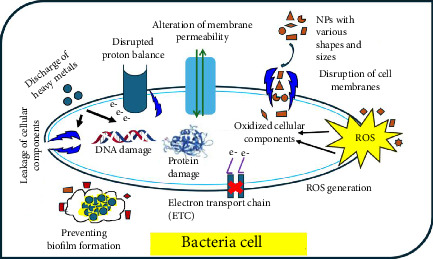
Illustrations of the various ways nanoparticles fight microbes.

ZnO NPs in suspension (infusion or emulsion) exhibited antimicrobial activity against Gram-positive bacteria, as shown in Table 2. Mechanistically, studies have shown that NP suspensions exhibit significant antibacterial efficacy against Gram-positive bacteria due to their ability to easily penetrate the bacterial core and provide a considerable interaction surface area, thereby hindering the growth mechanism (Figure 6).

**Figure 7:**
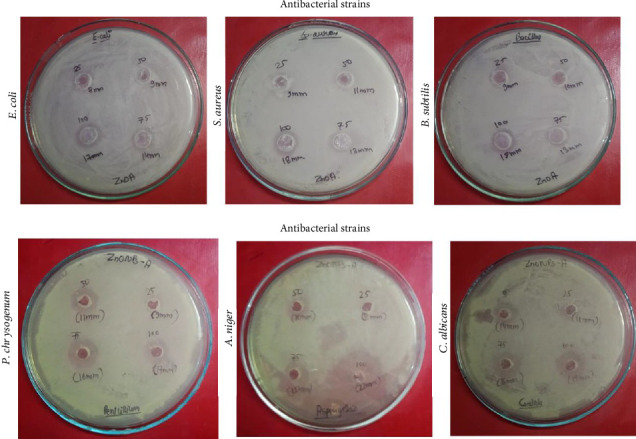
Antimicrobial activity of ZnO NPs showing zones of inhibition against *E. coli*, *S. aureus*, *B. subtilis*, *P. chrysogenum*, *A. niger*, and *C. albicans*.

Additionally, metal ions released from the NP surface may contribute to their antimicrobial properties [49]. Previous research has also shown that the antibacterial effect of metal oxide NPs and similar NPs depends on the presence of defects or oxygen vacancies on their surfaces, leading to lipid peroxidation and the generation of reactive oxygen species. In contrast, Leung et al. synthesized graphene oxide NPs and demonstrated a mode of microbial cell death independent of reactive oxygen species. In silico analysis, such as molecular docking, can be employed to investigate the interactions between NPs and potential receptor targets. This approach can elucidate key noncovalent interactions that contribute to the antimicrobial properties of NPs, including inhibition of bacterial growth or eradication of bacteria. To evaluate the antimicrobial efficacy of the synthesized ZnO NPs, tests were performed against a range of bacterial and fungal pathogens.  Figure 7 displays the IZ observed for ZnO against *E. coli*, *S. aureus*, *B. subtilis*, *P. chrysogenum*, *A. niger*, and *C. albicans*. The clear zones, varying in size, demonstrate ZnO's ability to suppress microbial growth, confirming its potent antimicrobial activity.

The antibacterial activity of ZnO NPs was thoroughly evaluated against Gram-negative *Escherichia coli* and Gram-positive *Staphylococcus aureus* and *Bacillus subtilis*, with streptomycin serving as the positive control for comparative analysis [50, 51]. The results consistently revealed a clear concentration-dependent antibacterial effect, with IZ increasing progressively from 25 to 100 mg/mL (Table 2).

Two key indicators were used in this evaluation: the IZ and the AI. The IZ is the transparent area around the antimicrobial on the agar plate that effectively inhibits bacterial growth. Its size is usually measured in millimeters (mm), which is directly related to the diameter of the transparent circle, indicating the effectiveness of the antimicrobial. The larger the IZ, the higher the activity. The AI quantifies the relative antimicrobial potency of a test sample (e.g., ZnO NPs) relative to a standard antimicrobial agent (e.g., streptomycin). It is calculated by dividing the IZ diameter of the sample by the IZ diameter of the standard. An AI value of 1.0 indicates activity comparable to that of the standard; a value less than 1.0 indicates a lower potency, and a value greater than 1.0 indicates higher potency. For *E. coli*, the IZ of ZnO NPs ranged from 8 mm at the lowest concentration to 17 mm at the highest dose tested, while the AI increased from 0.33 to 0.53. Although these values are still below the levels of streptomycin (24–32 mm), the antibacterial activity continued to rise with increasing NP concentration, suggesting a potential antibacterial mechanism. A similar dose-dependent response pattern was observed against Gram-positive strains. *Staphylococcus aureus* treated with ZnO NPs showed an IZ ranging from 9 to 18 mm, while *Bacillus subtilis* showed comparable activity (9–18 mm), although the AI was slightly lower (0.29–0.47 for *S. aureus* and 0.29–0.43 for *B. subtilis*). At the same concentration, the activity against *S. aureus* was somewhat higher than that against *E. coli*, which may be due to the structural differences in the cell wall composition of Gram-positive and Gram-negative bacteria. The thicker peptidoglycan layer characteristic of Gram-positive bacteria may make them more susceptible to NP-mediated destruction.

**Figure 8:**
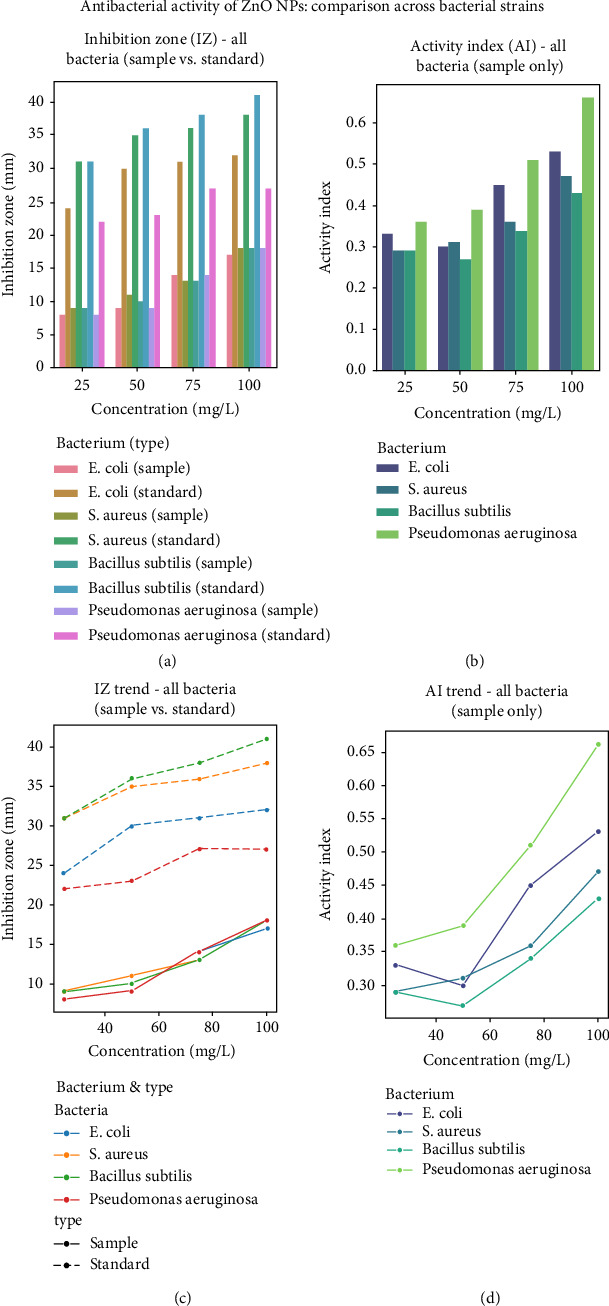
Antibacterial activity of ZnO NPs: Comparison across bacterial strains. (a) Inhibition zone (IZ) values for ZnO NPs (Sample) and streptomycin (Standard) against various bacterial strains at increasing concentrations. (b) Activity index (AI) of ZnO NPs against each bacterial strain. (c) Dose-response trends of IZ for ZnO NPs (Sample) and streptomycin (Standard) against all bacterial strains. (d) Dose-response trends of AI for ZnO NPs against all bacterial strains.

The antibacterial activity of ZnO NPs against various bacterial strains was comprehensively analyzed as shown in Figure 8 (Figures 8(a), 8(b), 8(c), 8(d)) and Table 2. Figure 8(a) shows the IZ bar graph comparing the IZ of ZnO NPs (samples) and streptomycin (standard) against all bacterial strains at increasing concentrations, where the IZ produced by ZnO NPs was consistently less than the standard but showed an apparent concentration-dependent increase in efficacy. Figure 8(b) illustrates the AI bar graph for ZnO NPs alone, reflecting an overall upward trend across all strains with increasing concentration, indicating an increase in relative antibacterial performance. Figure 8(c) is an IZ trendline graph depicting the concentration-dependent IZ progression for the samples and standards, with streptomycin consistently maintaining a higher IZ while ZnO NPs showed a steady improvement. Figure 8(d) shows the AI trendline graph for ZnO NPs, indicating a significant positive correlation between NP concentration and AI values for all bacterial strains. Together, the results in Figure 8 highlight that ZnO NPs exhibit broad-spectrum antimicrobial activity against both Gram-positive and Gram-negative bacteria, which may be mediated through nonspecific mechanisms, such as membrane disruption, reactive oxygen species generation, and Zn^2+^ ion release [52–54]. Although the potency of ZnO NPs was lower than that of streptomycin (all AIs < 0.53), the observed concentration-response pattern confirms its potential for optimization. These findings suggest promising applications in biomedical fields, including wound dressings, medical coatings, and adjunctive antimicrobial therapies. Further studies are needed to elucidate the mechanism of action, optimize the formulation, explore synergy with antibiotics, and evaluate the cytotoxicity and environmental safety of ZnO NPs to determine their potential as viable antimicrobial agents.

**Figure 9:**
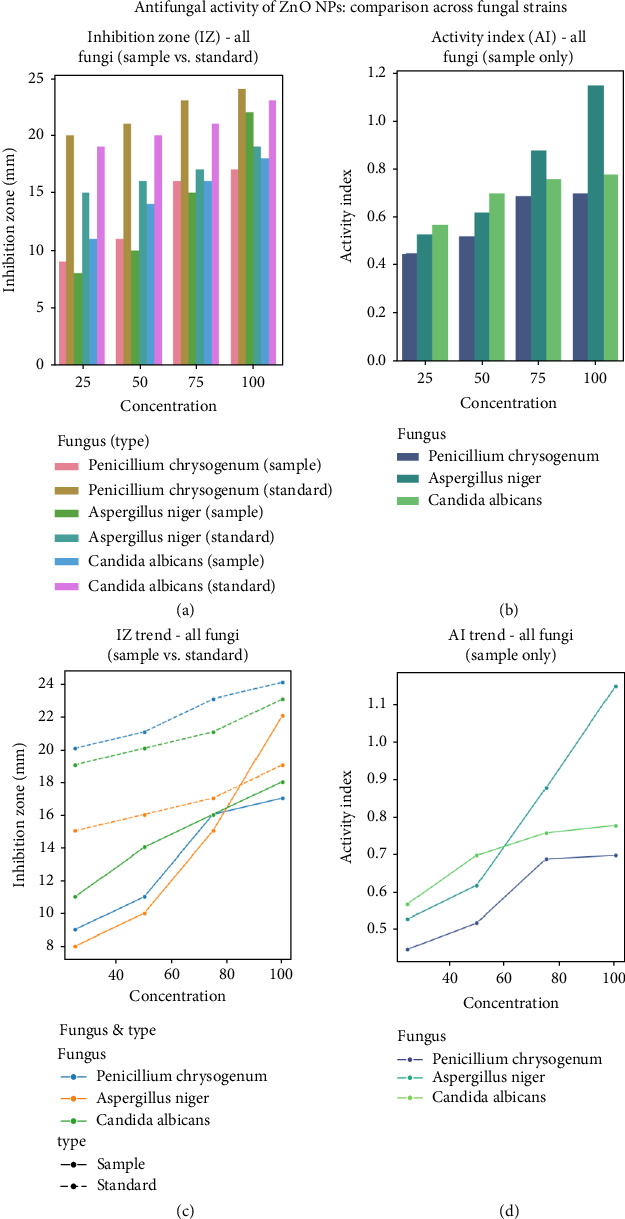
Antifungal activity of ZnO NPs: Comparison across fungal strains. (a) Inhibition zone (IZ) values for ZnO NPs (Sample) and ketoconazole (Standard) against various fungal strains at increasing concentrations. (b) Activity index (AI) of ZnO NPs against each fungal strain. (c) Dose-response trends of IZ for ZnO NPs (Sample) and ketoconazole (Standard) against all fungal strains. (d) Dose-response trends of AI for ZnO NPs against all fungal strains.

The antifungal activity of ZnO NPs against three fungal strains *(P. chrysogenum, A. niger*, and *C. albicans*) was evaluated and compared with the standard antifungal drug ketoconazole. The results showed that ZnO NPs showed a clear concentration-dependent response against all tested fungal species (Table 2). For *P. chrysogenum*, the IZ expanded from 9 mm at 25 mg/mL to 17 mm at 100 mg/mL, and the corresponding AI increased from 0.45 to 0.70. A similar trend was observed for *A. niger*, where the IZ expanded from 8 mm to 22 mm, and it is noteworthy that the AI reached 1.15 at the highest concentration, indicating a particularly strong inhibitory effect at 100 mg/mL. For *C. albicans*, the IZ ranged from 11 mm to 18 mm, and the AI increased from 0.57 to 0.78 over the concentration gradient. Compared with ketoconazole, the standard antifungal drug showed a larger IZ at all concentrations, which is consistent with the expectation of a clinically proven drug. However, ZnO NPs showed good activity, especially at higher concentrations, with a particularly strong effect against *A. niger*, as the AI exceeded 1.0 at 100 mg/mL. This suggests that while ketoconazole remains more effective, ZnO NPs may exert their antifungal effects through a different mechanism, possibly involving NP-specific interactions, such as the generation of reactive oxygen species or the gradual release of zinc ions, which can disrupt fungal cell membranes or metabolic pathways. The dose-dependent response of ZnO NPs and ketoconazole indicated that increasing concentrations enhanced the antifungal efficacy, but higher doses of the NPs were required to achieve an effect comparable to the standard.  Figure 9 shows the antifungal activity against three strains, with all fungal data combined into four different panels for easier comparison.  Figure 9 illustrates the antifungal activity of ZnO NPs by comparing their effects in different fungal strains.  Figure 9(a) (IZ Bar Graph; All Fungi (Samples vs. Standards)) compares the IZ values of ZnO NPs (samples) and ketoconazole (standards) at increasing concentrations in all fungal strains.  Figure 9(b) (AI Bar Graph; All Fungi (Samples Only)) shows the calculated AI of ZnO NPs for each fungal strain.  Figure 9(c) (IZ Trend Line Graph; All Fungi (Samples vs. Standards)) illustrates the IZ dose response trend for all fungal strains, distinguishing between samples and standards.  Figure 9(d) (AI Trend Line Graph-All Fungi (Samples Only)) depicts the AI dose response trend for ZnO NPs against all fungal strains. AI values, especially those approaching or exceeding 1.0 for ZnO NPs against *A. niger*, highlight their potential as an alternative or adjuvant to traditional antifungal drugs. The increased effectiveness at higher amounts may be due to the special features of the NPs, such as their large surface area and the way they slowly release active ingredients, which could disrupt the fungus's enzyme systems or cell wall structure. However, these results are from laboratory tests, and further research is needed to understand how effectively ZnO NPs work in organisms, their safety, and how they interact synergistically with current antifungal drugs [55]. Such studies may provide recommendations for overcoming fungal resistance and improving treatment outcomes. Overall, the results suggest that ZnO NPs are highly effective against fungi, primarily when used in large quantities. Further research is needed to improve their use and investigate their effectiveness in real medical situations.

**Figure 10:**
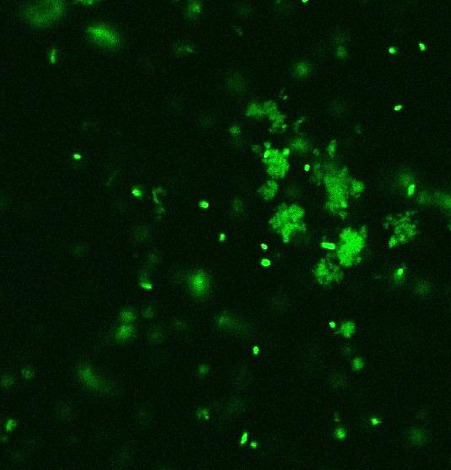
CLSM image of a bacterium with internalized nanoparticles (bright green dots).

The study also used confocal laser scanning microscopy (CLSM) to examine the cellular uptake of NPs and their interaction with *B. subtilis*. Understanding these interactions is critical to creating advanced treatments, including NP-based drug delivery systems to treat fungal and bacterial infections. CLSM images provide a comprehensive view of the cellular context, as shown in Figure 10. Confocal displays a network of fungal cells stained with fluorescent dye. Interestingly, the injected NPs dispersed throughout the cells as bright green spots. This means that the NPs may be interacting with internal cellular components because they were able to pass through the bacteria's cell wall. These interactions of NPs with the cell walls of microbial species can inhibit their growth in various ways, as shown in Figure 10.

### 3.3. Molecular Docking Analysis

**Figure 11:**
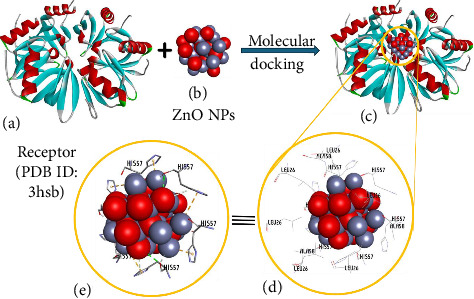
Depicts the results of molecular docking analysis showing interactions: (a) between receptor molecules and nanoparticles, (b) between nanoparticles themselves, (c) nanoparticles located within cavities, highlighted with interactions of different reactive amino acid residues, (d) illustrate various nonbonded interactions, and (e) monitor nonbonded interactions involving nanoparticles.

The interaction between the YmaH (Hfq) crystal structure of *Bacillus* subtilis (PDB ID: 3HSB) and ZnO NPs (Figures 11(a) and 11(b)) was studied using the online platform Webina equipped with AutoDock Vina. The docking results (Figure 11) were analyzed to determine the most favorable binding pose based on strong negative binding energy. The model with the lowest binding energy was selected for further analysis of amino acid interactions. Key residues (LEU26, ALA58, and HIS57) of all five chains of the protein (A, B, C, D, and E) were observed clustered around the zinc atoms of the NPs (Figures 11(c), 11(d), 11(e)). Notably, the HIS57 residues of each chain are involved in hydrogen bonding with the NPs. Furthermore, these HIS57 residues and other interacting amino acids may undergo nonbonded pi-cation interactions with Zn atoms. The different orientations of these residues suggest interactions with varying atoms of zinc on the NP surface. These findings highlight these amino acids as potential targets for the inhibitory effects of NPs. Furthermore, the interacting residues may also contribute to the electrostatic attraction with the Zn atoms present in the NPs.

### 3.4. Assessment of Photocatalytic Activity

**Figure 12:**
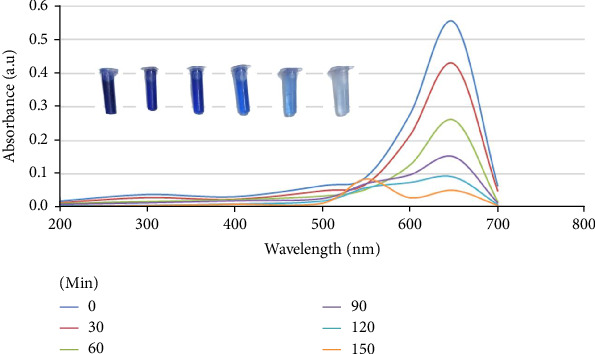
UV–vis absorbance spectra showing the photocatalytic degradation of MB dye by bioreduced ZnO NPs under sunlight irradiation, with corresponding visual changes in the solution's color shown in the inset test tubes.

**Figure 13:**
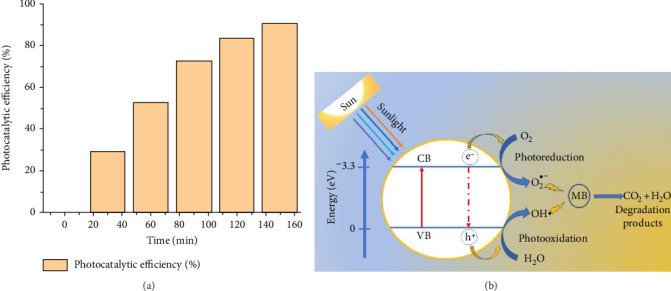
(a) Plot showing the increase in photocatalytic efficiency (%) of ZnO NPs with increasing irradiation time (minutes) during the degradation of methylene blue, (b) Photocatalytic degradation mechanism of Methylene Blue by Biogenic ZnO NPs (VB = valence band and CB = conduction band).

The degradation profile of MB dye under sunlight by bioreduced ZnO NPs catalyzed is given in Figures 12 and 13. The characteristic absorbance wavelength of the MB dye, observed at λmax = 665 nm, showed a decrease corresponding to the increasing reaction time, ranging from up to 150 min, in the presence of the ZnO NPs catalyst. Figures 12 and 13 display the photocatalytic degradation of MB dye by ZnO NPs under simulated solar irradiation. The photocatalytic efficiency, expressed as a percentage, is plotted against irradiation time, ranging from 0 to 150 min.  Figure 12 presents a series of UV–vis spectra that monitors the photocatalytic degradation of MB dye by ZnO NPs under simulated solar irradiation. The spectra, recorded at time intervals of 0, 30, 60, 90, 120, and 150 min, reveal a gradual decrease in the intensity of the characteristic MB peak centered around 665 nm. This decrease in absorbance directly correlates with the diminishing concentration of MB dye in the solution, providing compelling evidence for its successful degradation by the ZnO NPs. Concomitantly, subtle spectral shifts or the emergence of new peaks might be observed, indicating the potential formation of intermediates or byproducts during the photocatalytic process. Overall, this series of spectra effectively illustrates the dynamic progress of MB dye degradation by ZnO NPs, providing valuable insights into the reaction kinetics and the effectiveness of the photocatalytic system.

The results demonstrate a clear trend of increasing photocatalytic efficiency with extended irradiation time. At the onset of irradiation (0 min), the photocatalytic efficiency is naturally zero. However, within 30 min, a significant increase in photocatalytic efficiency to 29% is observed. This rapid initial increase suggests that the ZnO NPs are quickly activated by the light and begin to catalyze the degradation of MB. The efficiency continues to rise steadily, reaching 52.7% at 60 min and 72.46% at 90 min. Beyond 90 min, the rate of increase slows down slightly, with the efficiency reaching 83.33% at 120 min and 90.4% at 150 min. The observed trend can be attributed to several factors. Initially, the high concentration of MB molecules provides ample substrate for the photogenerated electron-hole pairs on the ZnO NPs to react with. As the reaction progresses, the MB concentration decreases, leading to a slower rate of degradation (Figure 13(a)). Additionally, the accumulation of reaction intermediates or the formation of byproducts might also influence the reaction and affect the overall photocatalytic efficiency. As depicted in Figure 13(b), under sunlight irradiation, ZnO NPs generate electron-hole pairs. These charge carriers react with adsorbed oxygen and water to produce highly reactive hydroxyl radicals (OH). The OH radicals generated then oxidize the adsorbed MB dye molecules, leading to their degradation into degradation products.

## 4. Conclusion

The present study introduces an innovative, environmentally friendly synthesis technique for producing ZnO NPs using an extract from *Spirodela polyrhiza*. This method offers a new approach in NP synthesis by leveraging natural materials for efficient production. The synthesized ZnO NPs demonstrated significant antibacterial efficacy against harmful microorganisms. Initial results suggest that higher doses of these NPs may also exhibit potential antifungal activity against *Candida albicans*; however, further research is required to validate this effect and refine treatment protocols. Among the tested fungal strains, *Aspergillus niger* showed the highest sensitivity, with an IZ of 22 mm and an AI exceeding 1.0 at 100 mg/mL, suggesting strong potential for antifungal applications. Future investigations should focus on elucidating the antibacterial mechanisms of ZnO NPs by exploring their interactions with bacterial proteins. Specifically, molecular docking simulations should be employed to identify critical amino acid residues involved in these interactions. Additionally, optimizing production techniques to enhance the stability and performance of these biogenic ZnO NPs will be crucial for maximizing their potential applications. Moreover, the findings from the photocatalytic experiments demonstrate that ZnO NPs are proficient in degrading MB dye when exposed to sunlight. A remarkable degradation efficiency of 90.4% was recorded after 150 min of irradiation, underscoring the promise of this system for environmental cleanup. In the future, integrating these biogenic NPs into composite materials or smart delivery platforms could further expand their utility in clinical and environmental fields.

## Figures and Tables

**Table 1:** Comparison of ZnO nanoparticle properties synthesized by different aquatic plant species.

Aquatic plants	UV–vis absorption peak/range (nm)	Size and shape	Antimicrobial activity	Ref.
*Echinochloa colona (E. colona) plant*	360–370 nm	Spherical and hexagonal 15.8 nm	Antibacterial	[26]
Sea Lavender *(Limonium pruinosum* L*. Chaz)*	370 nm	Hexagonal/cubic crystalline ∼ 41 nm	Antimicrobial and antioxidant	[27]
*Nelumbo nucifera* (lotus)	380 nm	Spherical 3–4 nm	—	[28]
*Lemna minor*	369 nm	Nano-Lamellar structure 20–100 nm		[29]
Water Hyacinth (*Eichhornia crassipes*)	270 nm	Spherical 24–36 nm	—	[30]
Rosemary		Rod and flower 88.87 nm	Antibacterial activity	[30]
Algal	370 nm	Crystalline hexagonal wurtzite structure 18 and 50.32 nm	—	[31]
*Spirogyra hyalina*	358 nm	Spherical 65 nm	Antibacterial	[32]
*Spirodela polyrhiza*	∼349 nm	Spherical and nonspherical 20–70 nm	Present	

**Table 2:** Antibacterial and antifungal activity of ZnO NPs compared to standard drugs (streptomycin for bacteria, ketoconazole for fungi). Shows inhibition zone (IZ, in mm) and activity index (AI) at varying concentrations.

Antibacterial activity	Conc. (mg/mL)	*E. coli*		*S. aureus*		*B. subtilis*	
		IZ	AI	IZ	AI	IZ	AI
ZnO NPs	25	8	0.33	9	0.29	9	0.29
	50	9	0.30	11	0.31	10	0.27
	75	14	0.45	13	0.35	13	0.34
	100	17	0.53	18	0.47	18	0.43

Streptomycin (Standard)	25	24		31		31	
	50	30		35		36	
	75	31		36		38	
	100	32		38		41	

**Antifungal activity**	**Conc. (mg/mL)**	***P. chrysogenum***		***A. niger***		***C. albicans***	
		**IZ**	**AI**	**IZ**	**AI**	**IZ**	**AI**

ZnO NPs	25	9	0.45	8	0.53	11	0.57
	50	11	0.52	10	0.62	14	0.70
	75	16	0.69	15	0.88	16	0.76
	100	17	0.70	22	1.15	18	0.78

Ketoconazole (Standard)	25	20		15		19	
	50	21		16		20	
	75	23		17		21	
	100	24		19		23	

## Data Availability

The data that support the findings of this study are available from the corresponding authors upon reasonable request.
